# Modulation of microbial communication by lipid exudates

**DOI:** 10.1093/ismejo/wrag156

**Published:** 2026-06-16

**Authors:** Mahmoud Gargouri, Philip D Bates, Stéphane Declerck

**Affiliations:** Earth and Life Institute, Applied Microbiology, Mycology, Université catholique de Louvain, Croix du Sud 2, Box L7.05.06, Louvain-la-Neuve 1348, Walloon Brabant, Belgium; Institute of Biological Chemistry, Washington State University, Pullman, WA 99164, United States; Earth and Life Institute, Applied Microbiology, Mycology, Université catholique de Louvain, Croix du Sud 2, Box L7.05.06, Louvain-la-Neuve 1348, Walloon Brabant, Belgium

**Keywords:** root exudates, lipidomics, quorum sensing, arbuscular mycorrhizal fungi, hyphosphere, synthetic microbial communities

## Abstract

Root exudates comprise a diverse mixture of nonpolar and amphiphilic compounds that are only partially recovered by aqueous extraction methods, yet can rival polar metabolites as carbon sources for microorganisms. Because many quorum sensing (QS) signals are fatty-acyl derivatives, lipid-rich microhabitats at the root–soil interface are likely to influence signal partitioning, persistence, and local QS thresholds. We propose a lipid-mediated framework in which plant-derived lipids modulate QS through four nodes: receptor mimicry/antagonism, quorum quenching, membrane/microenvironment regulation, and lipid-dependent resource gating. These mechanisms operate across two lipid-rich interfaces—the rhizosphere and the arbuscular mycorrhizal fungi hyphosphere—where host lipid fluxes may restructure microbial community composition. Combined with QS-sensitive changes in root exudation, this spatially structured lipid circuit could generate feedbacks influencing microbiome composition and function, with potential implications for microbiome engineering.

## Introduction

Root exudates are a key pathway by which plants influence the abundance, activity, and spatial organization of microorganisms in the rhizosphere. Most work on microbiome assembly via exudates has focused on polar metabolites, and large-scale metabolomics has shown that exudate composition varies with plant developmental stage, genotype, and environmental conditions, with measurable consequences for microbiome structure and function [1–4]. Metabo-lipidomics now adds another layer to this picture by showing that roots also release amphiphilic and hydrophobic compounds whose contribution to rhizodeposited carbon can be substantial, and in some systems, equal or even exceed that of the polar fraction [5]. These compounds should not be viewed only as nutrients. Indeed, many lipids partition into interfaces, modify membrane properties, and undergo oxidation, remodeling, or selective retention. They can therefore influence the physicochemical context of microbial interactions [6].

Lipid exudation should not be assumed to be uniform across plants. More generally, root exudate composition varies widely among plant species and genotypes, across developmental stages, and in response to environmental conditions; it is further influenced by microbial status and methodological differences in sampling [7–11]. The same caution applies to the hydrophobic fraction. Although recent metabo-lipidomics indicates that roots can release substantial amounts of amphiphilic and hydrophobic compounds [5], the extent and prevalence of this phenomenon across plant taxa remain insufficiently resolved, largely because lipid-rich exudate fractions have been sampled far less systematically than polar metabolites. Accordingly, we do not consider lipid exudation a universal trait expressed at comparable levels across plants. Rather, we view it as a context-dependent feature whose functional significance is likely to emerge in specific taxa, developmental stages, and ecological settings.

One interaction system likely to be particularly sensitive to such context dependence is quorum sensing (QS), a density-dependent mode of microbial communication in which cells produce, release, detect, and respond to small diffusible molecules that accumulate locally with increasing cell density and residence time [12, 13]. In canonical bacterial systems, QS entails signal production, extracellular accumulation, receptor-mediated detection, and downstream transcriptional responses that coordinate collective behaviors such as biofilm formation, motility, exoenzyme secretion, secondary metabolism, and virulence [14, 15]. QS is not detected by bulk cell density *per se*, but by the interplay among signal production, stability, diffusion, and local retention within spatially structured microhabitats [16]. Accordingly, the relevant variable is not signal production alone, but the effective concentration, persistence, and spatiotemporal interpretation of signals.

Several QS systems are chemically and physically linked to lipids. In gram-negative bacteria, *N*-acyl-homoserine lactones (AHLs) contain variable fatty-acyl chains, whereas diffusible signal factor family signals are *cis*-2 unsaturated fatty acids [17]. Fungi likewise employ density-dependent small molecules, including lipid-derived mediators such as farnesol and oxylipins, to regulate morphological transitions and population-level behaviors [18]. In parallel, many plant-associated bacteria encode LuxR-family “solo” regulators that lack a cognate LuxI-type AHL synthase and can respond to non-self ligands, including plant-derived compounds, thereby providing a plausible receptor interface between host chemistry and microbial communication [19–24]. Consistent with this, plants can produce AHL-mimicking compounds that perturb LuxR-regulated behaviors, including QS-dependent biofilm phenotypes, in plant-associated bacteria [25, 26]. These observations do not imply that free plant fatty acids broadly mimic canonical QS ligands. Rather, they motivate a more targeted question: under which mechanistic scenarios might lipid-rich plant exudates and/or interfaces modulate microbial communication?

The consequences of lipid exudation for microbial communication are therefore also likely to be context dependent [27, 28]. Moreover, many plant species, including numerous crops, associate with arbuscular mycorrhizal (AM) fungi, whose extraradical hyphae extend host-derived carbon flux into the surrounding soil and establish an additional interface, the hyphosphere [29]. Because AM fungi acquire lipids from the host and redistribute carbon through their extraradical mycelium [30–33], the hyphosphere may constitute a spatially structured environment in which lipid availability, surface-associated growth, and microbial communication intersect. We emphasize, however, that this remains a hypothesis-generating extension of the framework rather than a demonstrated case of QS regulation along fungal hyphae.

**Figure 1:**
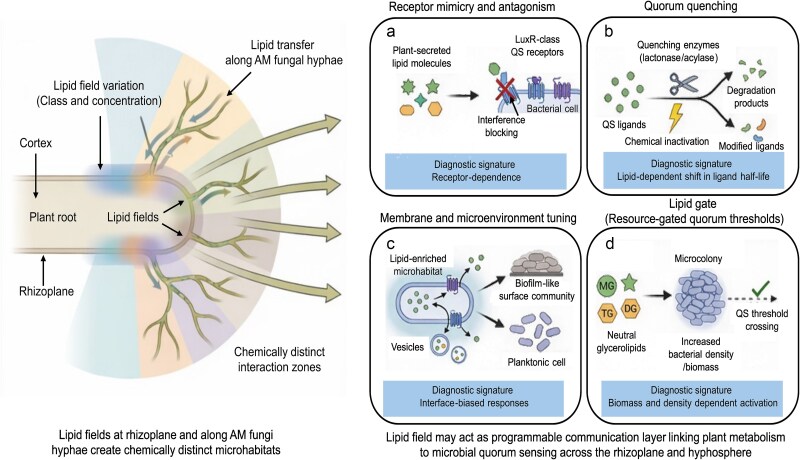
Lipid fields as a QS control layer at the root interface; left side: roots generate spatially structured lipid fields at the rhizoplane that vary both in lipid class and concentration; these fields can extend along AM fungal hyphae via lipid transfer, creating chemically distinct interaction zones beyond the root surface; within these lipid-enriched microhabitats, microbial QS outcomes are modulated through four separable control nodes; (a) receptor mimicry or antagonism: plant exudate lipids or lipid-derived metabolites can interact with QS receptors, including canonical LuxR proteins and LuxR solo regulators, modifying signal interpretation without necessarily altering ligand abundance, a diagnostic signature of receptor-dependent control; (b) quorum quenching: in lipid-enriched root and AM fungi-hyphal microhabitats, plant-exuded lipids tune the effective signal pool by shifting QS-ligand half-life (±) by altering ligand accessibility/partitioning and by modulating enzymatic quenching (lactonase/acylase) and/or oxidation/redox-driven inactivation thereby changing effective communication distance; diagnostic signature: a lipid-dependent change in ligand half-life with depletion of intact ligands and accumulation of modified and/or degradation products; (c) membrane and microenvironment tuning: lipid-mediated alterations in membrane properties, surface retention, and vesicle-associated transport reshape signal partitioning, uptake, and receptor context, producing more pronounced effects in surface-attached biofilms than in well-mixed liquid cultures, a diagnostic signature evident as interface-biased responses; (d) lipid gate (resource-gated quorum thresholds): pulses of neutral glycerolipids (MG/DG/TG) increase local carrying capacity and residence time within structured micro-colonies, enabling QS threshold crossing even in the absence of direct receptor binding, a diagnostic signature reflected in biomass- and density-dependent activation; collectively, lipid-rich interfaces may act as a modulatory layer linking plant metabolism, microbial growth conditions, and microbial communication across the rhizoplane and hyphosphere.

Here, we present a conceptual framework for how lipid exudates may modulate microbial communication across two connected plant-associated interfaces: the rhizosphere surrounding roots and the AM fungal hyphosphere associated with extraradical hyphae. Throughout, we explicitly distinguish direct evidence from mechanistic inference and testable hypotheses, avoid assumptions of adaptive intent, and treat QS as one diagnostic readout among several. We organize the framework into four nonexclusive nodes: receptor-level effects, ligand loss or protection, membrane/microenvironmental partitioning, and indirect resource-dependent threshold effects (Fig. 1). Thus, our framework does not presume that lipid–QS interactions are adaptive for the plant; rather, such effects may arise as emergent properties of spatially structured, lipid-rich microhabitats.

## Lipid rhizodeposition and interface formation

The strongest direct evidence currently comes from tall wheatgrass, where combined aqueous and organic extraction revealed lipid-derived carbon inputs approximately two-fold higher than those of polar metabolites, with markedly elevated C:N ratios [5]. Neutral glycerolipids (e.g. triacylglycerols) dominated the annotated hydrophobic fraction, arguing against passive leakage of membrane debris and instead suggesting selective enrichment of specific lipid classes. Additional observations from root-associated systems are consistent with regulated extracellular lipid release, including rapid incorporation of root-derived lipids and fatty acids into soil pools [34], the release of small extracellular vesicles from tomato roots into exudate solutions [8, 35], secretion of triacylglycerols into the apoplast in *Lithospermum erythrorhizon*, a root-associated system used to study extracellular export of lipophilic metabolites [36, 37], and the recovery of hydrophobic bioactive compounds from maize root exudation and root-surface washes [38]. Taken together, these studies support treating root-associated lipids and other hydrophobic exudate fractions as relevant, yet still under-characterized, components of rhizodeposition. The main lipid classes can be organized across the four QS control nodes (Fig. 1), together with representative biological systems and experimental approaches supporting each interaction (Table 1).

**Table 1:** Lipid classes mapped onto four QS control nodes.

Lipid class	Node	Best-supported QS intersection	Representative system/assay example	Evidence level	References
**Free fatty acids**/DSF-like fatty acids (C16–C18 unsaturated)	A/C	Fatty-acid chemistry is directly relevant in DSF-family signaling and may also influence signal behavior at interfaces	DSF-family systems tested with DSF-responsive mutants or reporters	Direct for DSF-family signaling; indirect for modulation by plant-derived fatty acids	[17, 36, 37]
**Oxylipins and other plant**-derived lipid-responsive metabolites	A	Plant-derived metabolites may alter how signals are interpreted through plant-responsive receptors or AHL-mimic activity	Rice and bean extracts affecting QS-regulated biofilm phenotypes; plant-responsive signaling in *Pseudomonas* sp. GM79	Direct for plant-responsive receptor or bioassay activity; contribution of lipid-derived compounds remains unresolved	[19–21, 25]
**Oxidized lipids**/reactive lipid species (lipid peroxides; reactive aldehydes)	B	Reactive lipid chemistry may shorten signal half-life or promote ligand inactivation	Ligand half-life assays and measurements of intact versus degraded ligand	Hypothesis-driven in plant lipid contexts	[20, 42]
**Membrane amphiphiles** (sterols; sphingolipids; lysophospholipids)	C	Amphiphiles may alter signal retention, permeability, and vesicle-associated transport	CAI-1 transport via outer membrane vesicles in *Vibrio harveyi*	Direct for interface-dependent transport of hydrophobic signals; indirect for similar effects of plant-derived amphiphiles	[39, 40]
**Neutral glycerolipids/AM fungal lipid redistribution**	C/D	Neutral lipids may alter signal partitioning at interfaces and increase local carrying capacity in lipid-rich microsites	Hypha-associated bacterial colonization in AM fungal systems; host-to-fungus lipid transfer	Direct for host-to-fungus lipid transfer and hypha-associated bacterial colonization; hypothesis-driven for QS modulation	[30–33, 46, 47, 59, 61, 62, 66]

QS is used here as the diagnostic readout. Where direct reporter-based evidence is not yet available, the interaction is explicitly labeled as hypothesis-driven rather than established. Nodes: (A) mimicry/antagonism, (B) quenching, (C) membrane/microenvironment, and (D) lipid gate. Abbreviations: AHL, acyl-homoserine lactone; AM, arbuscular mycorrhizal; DSF, diffusible signal factor; QS, quorum sensing.

Methods used to sample the root exudate metabolome, including its lipid fraction, are crucial for interpretation. Solvent capture of exuded compounds is powerful, but it can disrupt root function and thus exudation. Furthermore, hydrophobic compounds are consistently under-sampled when using primarily aqueous solvents. Quantitative comparisons of exudate collection procedures show that the sampling protocol (duration, solution volume/root ratio, aeration, inhibitors) significantly modifies the apparent exudation rates and profiles [27]. Low-disturbance, *in situ* sampling is essential for building causal narratives. Time-resolved coated-blade solid-phase microextraction coupled to ambient mass spectrometry can track root-exudate dynamics with minimal perturbation [28], and extending it with calibrated hydrophobic coatings should improve capture of nonpolar exudates while reducing concerns about solvent-driven artifacts.

To remain mechanistic rather than encyclopedic, we treat lipid diversity functionally by asking how lipids contribute to each QS control point: acting as receptor ligands or modulators, shaping membranes and interfaces that constrain diffusion and persistence, or supplying local carbon that gates growth and collective behaviors in structured microhabitats. This framing also clarifies why standard aqueous exudate profiling is not equivalent to characterizing the “root exudate lipidome”: the same lipid-derived molecules can influence QS as a signal, a carrier, or a threshold-setting resource.

## Why lipid chemistry may alter quorum sensing

QS depends on the effective concentration, persistence, and interpretation of signal molecules within structured space rather than on cell number alone [16]. Lipid-rich environments can, in principle, influence this effective concentration in several nonexclusive ways. First, hydrophobic or amphiphilic compounds can alter the partitioning of QS ligands among aqueous films, membranes, particles, vesicles, and biofilm matrices, thereby changing residence time, diffusion distance, and local accumulation [20, 39–41]. Second, oxidized lipids or other reactive lipid-derived species may shorten ligand half-life, whereas retention within lipid-rich phases may in some contexts protect hydrophobic signals from rapid loss. Third, plant-responsive receptors such as LuxR solos raise the possibility that some plant-derived metabolites act at the level of signal interpretation rather than as canonical ligand mimics [19–26]. In this view, lipid-dependent modulation of QS is best considered as a set of distinct mechanistic possibilities rather than a single process.

This framework is organized into four nodes. Node A considers receptor mimicry or antagonism, in which plant-derived compounds alter signal interpretation without necessarily changing ligand abundance [19–26]. Node B considers ligand loss or protection, including quorum quenching and lipid-dependent changes in signal half-life [20, 39–45]. Node C considers interface partitioning and microenvironment tuning, in which membranes, vesicles, and lipid-rich phases alter signal retention, transport, and receptor context [20, 39–41]. Node D considers resource gating, in which lipid-rich microsites increase local carrying capacity and residence time, thereby promoting QS threshold crossing in structured populations [46, 47]. Together, these four nodes provide a practical framework for distinguishing receptor-level, ligand-level, interface-level, and ecological effects of lipid-rich plant-associated habitats.

Some free fatty acids can also act as antimicrobial or membrane-disruptive compounds, so growth and viability need to be measured alongside QS readouts when assigning mechanism [42, 43]. Plants can likewise respond to QS molecules, creating a reciprocal dimension in which microbial signaling may reshape the chemical environment that supports subsequent signaling. We return to this feedback below, because direct evidence that defined QS ligands remodel the hydrophobic fraction of root exudates remains limited [48–58].

### Node A: receptor mimicry and signal reinterpretation

Receptor mimicry or antagonism represents the most direct route by which plant-derived compounds can influence QS, because it acts at the level of signal interpretation rather than signal abundance (Fig. 1a). In this scenario, lipid-derived or lipid-associated metabolites do not need to reproduce the full chemistry of canonical QS ligands in order to alter QS output. Instead, they may interfere with receptor occupancy, receptor conformation, or downstream transcriptional responses, thereby shifting how microbial cells interpret their local signaling environment [19–26, 41]. This distinction is important because a change in QS-regulated behavior does not necessarily imply a change in ligand production or persistence; it may instead reflect altered perception of the same signal pool (Table 2).

**Table 2:** Experimental tests that distinguish the four nodes of lipid–QS coupling: Node A—interpretation control (mimicry/antagonism), Node B—pool control (quenching/lipid-tuned half-life), Node C—microenvironment control (interface context), and Node D—resource gating (“lipid gate”).

Node	Targeted perturbation	Representative example	What to measure	Diagnostic signature	References
**Node A**	Test receptor-dependent effects using receptor mutants and competing ligands	Rice/bean AHL-mimic bioassays affecting QS-dependent biofilms in *Sinorhizobium fredii* SMH12 and *Pantoea ananatis* AMG501; plant-responsive signaling in *Pseudomonas* sp. GM79	QS reporter output; QS-regulated gene expression; ligand abundance	QS output changes without a major change in measurable ligand abundance	[20, 25]
**Node B**	Test ligand degradation or protection using half-life assays, enzyme perturbations, and fresh-ligand rescue	AHL lactonase-based quorum quenching and anti-virulence workflows in plant-associated systems	Ligand half-life; intact ligand; degradation products; QS output	QS output changes together with a change in ligand persistence or degradation	[44, 45]
**Node C**	Compare surface-associated and planktonic growth and measure ligand partitioning across interface-associated fractions	CAI-1 trafficking via outer membrane vesicles in *V. harveyi*; membrane-signal interaction studies	Ligand distribution among aqueous, membrane, vesicle, and biofilm-associated fractions; spatial gradients; apparent QS threshold	Effects are strongest in interface-dominated habitats, even when total signal production is similar	[39, 40]
**Node D**	Add neutral glycerolipids during structured growth and test whether local biomass and clustering increase before QS activation	Efficiency-sensing and biofilm sociobiology frameworks linking local density, structured growth, and QS threshold crossing	Biomass; clustering; local density; QS activation; growth metrics	Increased local growth or persistence precedes QS activation under lipid-rich conditions	[46, 47]

Avoid labeling a phenotype as “QS inhibition” before separating pool control (ligand loss/shortened half-life) from interpretation control (receptor reconfiguration), to prevent mechanism misattribution. Abbreviations: AHL, acyl-homoserine lactone; QS, quorum sensing.

The strongest support for this node comes from plant-responsive receptor systems and from plant-derived compounds that mimic or interfere with AHL-regulated behaviors. LuxR-family “solo” regulators are particularly relevant in this context because they lack a cognate LuxI-type synthase and are therefore positioned to respond to non-self ligands, including host-derived compounds [19–25]. Consistent with this, plants can produce metabolites that perturb LuxR-regulated phenotypes, including QS-dependent biofilm behaviors in plant-associated bacteria [25, 41]. These observations support the idea that some plant-derived compounds act at the level of signal interpretation, even when their chemistry differs from canonical acyl-homoserine lactones.

Current evidence supports receptor-level cross-talk in selected systems, particularly where plant-responsive LuxR-type receptors or related signal-responsive architectures are present [19–26]. The strongest case is therefore not for broad substitution of canonical AHL ligands by simple plant fatty acids, but for more selective modulation of receptor behavior by lipid-derived plant metabolites. Because the homoserine lactone moiety remains central to canonical AHL recognition, this node is best interpreted as a candidate host-associated signaling interface whose breadth across plant-microbe systems remains to be defined [14, 19, 21–26].

Node A is also the one most easily confused with general toxicity. Some free fatty acids can compromise membrane integrity or suppress growth, and the resulting decline in QS-dependent phenotypes may reflect reduced viability rather than altered signal interpretation [42, 43]. For this reason, receptor-level effects should be inferred only when QS output changes without a corresponding decline in measurable ligand abundance, and when the phenotype depends on receptor identity or receptor state rather than simply on biomass reduction (Table 3). Experimental tests are therefore most convincing when they combine QS reporters with receptor mutants, competitive ligand assays, and direct measurements of ligand pools, allowing interpretation control to be separated from ligand depletion or nonspecific stress effects (Table 2).

**Table 3:** Readouts that help distinguish the four nodes of lipid–QS coupling.

Node	What the node changes	Diagnostic signature (what you should observe)	Best supporting test	What would argue against this node	References
**Node A**	Change in receptor-mediated signal interpretation	QS-regulated phenotypes change without a corresponding drop in measurable AHL or DSF abundance; some responses may affect only part of the QS regulon	Receptor mutants, competing ligands, and reporter assays that show receptor dependence	Ligand abundance or half-life drops strongly, or fresh ligand alone restores the phenotype	[13–15, 20, 41]
**Node B**	Change in ligand abundance, persistence, or degradation	Lower measurable ligand abundance, shorter ligand half-life, and/or accumulation of degradation products	Half-life assays; measurements of intact ligand and degradation products; rescue by blocking quenching or adding fresh ligand	QS output changes without detectable effects on ligand abundance or persistence	[44, 45]
**Node C**	Change in signal retention, transport, or exposure at interfaces	QS responses are strongest under surface-associated conditions; signal distribution differs among aqueous, membrane, vesicle, or biofilm-associated fractions	Fractionated ligand measurements and comparison of planktonic versus surface-associated growth	Similar QS responses in well-mixed and interface-dominated conditions with no evidence of altered partitioning	[39, 40]
**Node D**	Change in local carrying capacity, persistence, or threshold crossing	Increased local biomass or clustering precedes QS activation; lipid-rich microsites increase the likelihood of QS activation	Time-resolved measurements of biomass, clustering, and QS reporter output under structured growth	QS changes occur without increases in local growth, persistence, or clustering	[46, 47]

Node A—interpretation control (receptor mimicry/antagonism), Node B—pool control (quenching/lipid-tuned ligand half-life), Node C—lipid-rich membrane/microenvironment tuning (partitioning/transport physics), and Node D—lipid gate (resource-enabled threshold crossing). Abbreviations: AHL, acyl-homoserine lactone; DSF, diffusible signal factor; QS, quorum sensing.

### Node B: ligand loss, protection, and quorum quenching

Quorum quenching and lipid-dependent changes in ligand persistence define the second major route by which lipid-rich interfaces may alter QS (Fig. 1b). In this node, the central variable is not how signals are interpreted, but how long they remain available in an active form. QS output can decline because ligands are enzymatically degraded, chemically inactivated, or rendered less accessible to their receptors. Conversely, signaling may be prolonged when ligands are retained within lipid-rich phases, vesicles, or interfaces that reduce their effective loss from the local microhabitat [39, 40, 44, 45]. This node therefore focuses on changes in ligand half-life, accessibility, and depletion rather than on direct receptor mimicry.

The best-established component of this node is quorum quenching itself. Enzymatic degradation of AHLs by lactonases or acylases is widespread in plant-associated systems and has already been exploited to reduce QS-dependent pathogenicity in planta [44, 45]. Within the present framework, plant-derived lipids are not treated as passive carbon background but as factors that could shift the efficiency of ligand loss. One possibility is that lipid-rich microhabitats increase ligand depletion by promoting oxidative or redox-mediated inactivation, or by enhancing the accessibility of ligands to quorum-quenching enzymes. Alternatively, lipids may protect signals by partitioning them into hydrophobic phases, membranes, or vesicle-associated compartments, thereby reducing their exposure to degradation [39, 40]. The key prediction is therefore not simply that signaling decreases, but that ligand half-life changes in a lipid-dependent manner.

Compared with Node A, the eco-evolutionary interpretation of Node B is more likely to begin from physicochemical consequences of structured interfaces than from highly specific inter-kingdom recognition. Lipid-rich root and hyphal microhabitats could alter ligand persistence even in the absence of evolved host control, simply because oxidation state, phase partitioning, and enzyme access differ across microsites. At the same time, microbial communities associated with plants may evolve to exploit or counteract such environments, e.g. by producing stronger quenching capacity or by using vesicle-based protection of hydrophobic signals [39, 40, 44, 45]. In that sense, Node B may often reflect an emergent ecological property of lipid-rich habitats, while still providing a substrate on which more specialized adaptations could evolve.

Node B must also be distinguished carefully from general growth inhibition. If lipid exposure suppresses biomass, apparent QS downregulation may simply reflect fewer viable cells rather than true ligand depletion. For that reason, evidence for Node B is strongest when changes in QS output occur together with measurable shifts in intact ligand abundance, ligand half-life, or degradation products, and when these effects can be reversed by enzyme inhibition or fresh ligand addition (Tables 2 and 3). Experimental tests should therefore quantify both the signal pool and its breakdown products while monitoring growth and viability, so that genuine changes in ligand persistence can be separated from nonspecific antimicrobial effects [42–45].

### Node C: interface partitioning and microenvironment tuning

Lipid-rich interfaces may also alter QS by changing how signals partition, move, and persist within structured microhabitats rather than by directly modifying receptor logic or ligand abundance (Fig. 1c). In this node, the key mechanism is physical and spatial: hydrophobic or amphiphilic compounds can redistribute QS ligands among aqueous films, membranes, particles, vesicles, and biofilm matrices, thereby altering diffusion distance, local retention, uptake, and receptor context [39, 40]. As a result, the same total amount of signal may generate different biological outcomes depending on how strongly it is retained at an interface and how effectively it reaches cells within that interface.

The clearest support for this logic comes from systems in which hydrophobic autoinducers are trafficked or stabilized through membrane-associated compartments. Outer membrane vesicles can facilitate the transport of hydrophobic QS molecules, illustrating that delivery physics alone can shape signaling outcomes [39, 40]. This principle is especially relevant in plant-associated interfaces, where cells rarely experience well-mixed liquid conditions. Instead, they occupy surfaces, thin water films, extracellular matrices, and particulate microhabitats in which lipid-rich phases may enhance local retention and create sharper chemical gradients than would be expected in bulk solution [16, 39, 40]. Under these conditions, signal exposure is determined not only by production rate, but also by how interface properties shape effective transport.

Node C becomes particularly important when the framework is extended beyond the rhizoplane to the hyphosphere. AM fungal hyphae provide a second plant-associated interface in which host-derived lipids are redistributed and microbial colonization occurs on a structured biological surface [29–33, 59–63]. Recent work supports the view that extra-radical hyphae recruit bacterial assemblages and act as distinct microbial interfaces rather than passive extensions of the surrounding soil [29, 31, 32, 60, 64]. In parallel, bacterial attachment and biofilm formation on fungal hyphae are well documented, and mixed fungal-bacterial systems show that fungal scaffolds can influence microbial organization and signaling-associated behavior [58, 64, 65]. Within the present framework, the hyphosphere is relevant because it provides a biologically realistic setting in which interface-mediated partitioning, retention, and signal delivery are likely to differ from those at the rhizoplane. Direct demonstration of QS regulation along AM hyphae remains an important next step.

From an eco-evolutionary perspective, Node C is best viewed primarily as an interface effect. Lipid-rich surfaces, vesicles, and biofilm-associated matrices can modify signal behavior even without any requirement for adaptive host intent. At the same time, repeated exposure to such structured habitats may favor microbial traits that exploit them, including surface-associated growth, vesicle-mediated transport, or altered responsiveness to retained ligands [12, 39, 40, 58, 64]. In that sense, Node C may often reflect a physicochemical consequence of lipid-rich interfaces, while also providing a stable ecological setting in which more specialized microbial adaptations can emerge.

The experimental signature of Node C is not simply a change in QS-regulated phenotype, but rather a shift in signal behavior that is strongest under interface-dominated conditions. Evidence for Node C is strongest when QS responses differ between surface-attached and well-mixed growth despite similar total ligand production, or when ligands are measurably redistributed among aqueous, membrane, vesicle, or biofilm-associated fractions (Tables 2 and 3). Accordingly, the most informative tests will combine spatially resolved QS reporters with fractionated measurements of ligand partitioning and interface-associated lipid composition, allowing transport physics to be separated from receptor-level reinterpretation or outright ligand depletion.

### Node D: resource gating and threshold crossing

The fourth node differs from the preceding three because it is primarily ecological rather than receptor- or ligand-centered (Fig. 1d). In this scenario, lipid-rich interfaces do not need to alter QS signal chemistry directly in order to influence QS output. Instead, high-energy lipids can increase local carbon availability, carrying capacity, and residence time within structured microhabitats, thereby raising the probability that microbial populations reach the density and persistence required for QS threshold crossing [46, 47]. Under this view, lipids function as threshold-setting resources: they make it more likely that surface-associated micro-colonies remain metabolically active and spatially concentrated long enough for signaling to become effective.

The resource-gating logic of Node D is especially relevant in plant-associated habitats, where microbial cells are rarely distributed evenly and instead occupy surfaces, aggregates, and patchy nutrient-rich microsites [16, 46, 47]. If lipid exudates increase the local energetic value of such microsites, they may indirectly promote QS-regulated behaviors such as biofilm maturation, cooperative secretion, or antagonistic interactions simply by sustaining larger or longer-lived local populations. In this sense, Node D does not require lipids to mimic signals or to change receptor occupancy. The key causal link is between lipid availability and the ecological conditions that permit QS activation. This is why QS remains the diagnostic readout in this node, whereas the proximal mechanism is resource-enabled threshold crossing rather than signal reinterpretation or signal loss.

The relevance of this mechanism may be particularly strong in mycorrhizal systems. AM fungi redistribute host-derived lipids and carbon into extra-radical hyphal networks, thereby generating a second interface in which bacterial colonization occurs under structured, surface-associated conditions [29, 30, 32, 33, 59–63]. The strongest current support does not yet show direct QS activation on AM hyphae, but it does indicate that hypha-associated bacterial communities are reproducible, spatially organized, and exposed to a distinct carbon landscape shaped by fungal transport and assimilation processes [29, 31, 32, 59–64]. Within the present framework, this makes the hyphosphere a plausible setting for resource gating, because lipid-enriched hyphal microsites could sustain local bacterial persistence and clustering in ways that make QS threshold crossing more likely than in the surrounding bulk soil. The same reasoning applies at the rhizoplane, where hydrophobic exudate patches and root-associated interfaces may create short-range, carbon-rich niches that disproportionately support surface-attached populations.

From an eco-evolutionary perspective, Node D is the most parsimoniously interpreted as an emergent ecological consequence of lipid-rich interfaces rather than as evidence of communication-specific host control. Lipids may reshape QS simply because they alter the resource landscape in which signaling occurs. At the same time, repeated selection in such environments could favor microbial traits that exploit resource-rich structured habitats, including tighter aggregation, altered threshold sensitivity, or more efficient investment in QS-regulated collective behaviors [46, 47]. Node D therefore provides perhaps the clearest example of how incidental metabolic effects could become ecologically consequential, and potentially, over longer time scales, evolutionarily elaborated.

The experimental signature of this node is distinct from those of Nodes A–C. Evidence for Node D is strongest when increased biomass, clustering, or local persistence precede QS activation, and when QS gating responds to additions of neutral lipids even though receptor chemistry and measurable ligand integrity remain unchanged (Tables 2 and 3). Conversely, if QS-related phenotypes shift without corresponding changes in local growth or clustering, then receptor-level reinterpretation, ligand depletion, or interface-mediated partitioning provide more likely explanation. The most informative tests will therefore combine time-resolved QS reporters with measurements of biomass, spatial aggregation, and lipid accessibility under structured growth conditions, allowing resource-enabled threshold crossing to be distinguished from the other three nodes.

## Hyphosphere as a second lipid-rich interface

AM fungal hyphae extend the present framework beyond the rhizoplane by providing a second plant-associated interface in which host-derived carbon and lipids are redistributed through extra-radical mycelium [29–33, 59, 61–63]. Because AM fungi sustain carbon flux along extra-radical hyphae, the hyphal surface may create localized microsites in which bacterial attachment, persistence, and signal retention differ from those at the rhizoplane [59, 61–63]. This makes the hyphal surface, within the broader hyphosphere, a plausible setting in which lipid-dependent effects on microbial communication may differ from those operating at the rhizoplane.

The strongest current support does not yet demonstrate direct QS regulation on AM fungal hyphae. Instead, it shows that extra-radical hyphae create reproducible, spatially structured microbial interfaces that recruit distinct bacterial communities and support growth [29, 60, 61, 64, 65]. Within the hyphosphere as a second lipid-rich interface framework, hyphae may alter microbial communication in at least two ways. First, they may amplify Node C by creating interface-dominated microsites in which membranes, surfaces, vesicles, and thin water films modify signal retention and delivery. Second, they may amplify Node D by redistributing host-derived carbon and lipids into localized hyphal microsites that promote bacterial persistence, aggregation, and threshold crossing [29–33, 59–64].

Whether AM fungi chemically transform host- or soil-derived lipids into forms directly available to associated bacteria *in situ* remain unresolved [63]. The more conservative interpretation is therefore that hyphae act primarily as structured biological interfaces whose topology, carbon flux, and associated microbial organization create conditions under which lipid-dependent modulation of QS becomes more likely. This interpretation is also consistent with observations that bacterial attachment, biofilm formation, dispersal, and contact-dependent interactions can all be facilitated on fungal hyphae [30, 64, 65]. Recent work further suggests that fungal-associated growth can itself be responsive to quorum-related regulation, because mixed fungal-bacterial biofilms show changes in architecture and adhesin expression in response to quorum signals [58]. Taken together, these findings support the hyphosphere as a biologically distinct, lipid-influenced interface that is particularly relevant to Nodes C and D, while direct evidence for communication-specific effects of fungal lipid transformation remains an open question requiring targeted experiments.

### Reciprocal feedbacks: microbial signals may reprogram plant lipid production

Plants respond to bacterial QS molecules with defense- and stress-associated transcriptional and metabolic changes. This indicates that microbial signaling can influence host physiology beyond direct effects on microbial communication [48–56]. Several studies have shown that AHL exposure activates salicylic-acid-, jasmonate-, and oxylipin-linked pathways or primes plants for stronger subsequent defense responses [48–56]. More broadly, microbial colonization and microbial community composition can reshape root exudation profiles and thereby alter the surrounding rhizosphere. Pathogen invasion can shift exudation patterns in ways that indirectly remodel the soil microbiome [59]. Stress-remodeled root exudates can stimulate and attract beneficial fungi such as *Trichoderma* [51], and metabolomic work in poplar shows that microbial colonization can rapidly and substantially rewire both the composition and content of root exudates [57]. Together, these observations support the broader idea that plant-released chemistry is dynamically reconfigurable in response to microbial cues.

The possibility that microbial cues reshape host exudation chemistry is relevant to the present framework because changes in exudation could, in principle, feed back onto the same lipid-rich interfaces that shape QS. Current evidence shows that microbial presence, microbial community composition, and defense activation can remodel host exudation chemistry, but whether defined QS ligands specifically and reproducibly alter the hydrophobic fraction of root exudates in a way that feeds back on QS itself remains to be tested directly [48–56]. We therefore treat this process as a plausible reciprocal extension of the framework rather than as a fully resolved mechanism.

A tomato split-root metabolomics study provides an informative example of community-driven remodeling of host exudation chemistry at the community level [52]. Distinct rhizosphere communities induced systemic changes in tomato root exudation, including features linked to acyl sugars and azelaic-acid-related metabolites [52]. These results show that microbial communities can reshape host exudation chemistry at a distance, but they are more parsimoniously interpreted as defense- or community-associated metabolic remodeling than as evidence of communication-specific recruitment. The key unresolved question is therefore not whether microbial cues alter plant exudation, but whether the hydrophobic component of these changes can feed back on QS through any of the four nodes described above. Answering that question will require experiments that combine defined QS ligands or QS-active communities with direct quantification of the hydrophobic exudate fraction, alongside spatially resolved QS readouts at root- and hypha-associated interfaces.

### Engineering opportunities and failure modes

If lipid-rich interfaces modulate QS in reproducible ways, they may provide useful entry points for microbiome engineering. Because receptor modulation, ligand loss or protection, interface partitioning, and resource gating act through different mechanisms, they also point to different translational strategies. Receptor-level effects (Node A) suggest the possibility of designing lipid-inspired compounds that interfere with signal interpretation. Ligand-centered effects (Node B) point to anti-virulence strategies that accelerate signal loss or prevent signal protection without relying on bactericidal activity [44, 45]. Interface-mediated effects (Node C) raise the possibility of manipulating retention, transport, or surface-associated signaling environments; whereas resource-gating effects (Node D) suggest that host genotypes or engineered exudation profiles can reshape the local ecological conditions under which QS activation becomes more or less likely.

These node-specific translational possibilities also extend to host-mediated intervention. Breeding or engineering plants to alter the composition, abundance, or ratio of exuded lipids could, in principle, shift microbial cooperation, antagonism, colonization, or pathogen suppression at the rhizoplane and hyphosphere. A complementary strategy would be to design synthetic microbial communities whose QS programs are activated only under defined lipid conditions, thereby converting host-associated chemistry into an input layer for engineered community behavior. However, the feasibility of such approaches depends on distinguishing clearly among the four nodes, because similar phenotypic outcomes may arise from very different underlying mechanisms.

The major failure mode is that the same lipid environments that suppress one microbial function may enhance another. In particular, pathogens may exploit lipid-rich microsites to accelerate virulence, stabilize biofilms, or gain earlier access to QS activation thresholds, a risk we refer to here as “QS hijacking.” For this reason, translational work will require careful context mapping across host genotype, microbial community composition, stress regime, and soil structure. In practice, engineering efforts are most likely to succeed when they are grounded in node-specific mechanisms rather than in the general assumption that increasing or decreasing lipid exudation will predictably improve microbiome function.

## Conclusions and future directions

In conclusion, the four-node framework proposed here identifies four mechanistic routes by which lipid-rich interfaces may alter microbial QS: receptor mimicry or antagonism (Node A), ligand loss or protection (Node B), interface partitioning and microenvironment tuning (Node C), and resource-dependent threshold crossing (Node D). Across these nodes, hydrophobic and amphiphilic compounds released at root-associated interfaces, and potentially redistributed along AM fungal hyphae, may reshape the physicochemical and nutritional conditions under which microbial communication occurs. In this sense, lipid-dependent effects on QS are best viewed as context-dependent properties of spatially structured microhabitats whose functional significance remains to be established experimentally.

**Figure 2:**
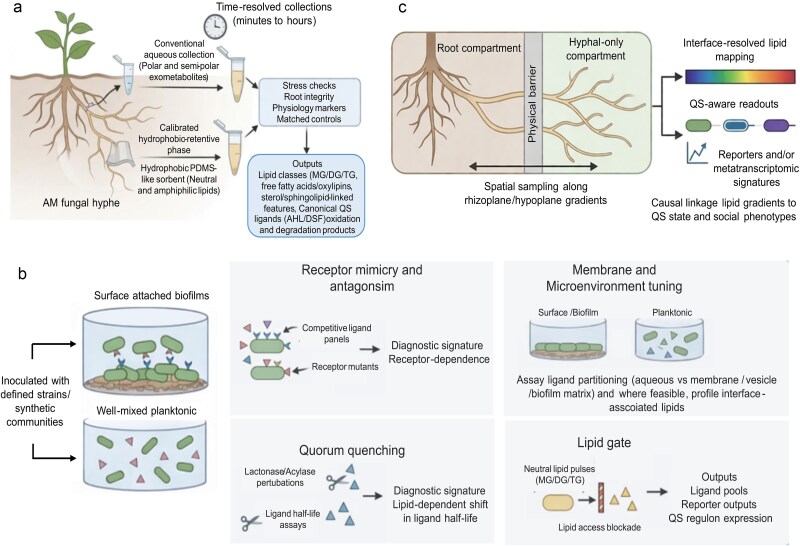
Experimental designs to test lipid–QS causality; (a) paired aqueous–hydrophobic sampling with stress controls; a chemically inclusive exudate workflow combines conventional aqueous collection, which captures polar and semi-polar metabolites, with a calibrated hydrophobic-retentive phase (e.g. passive sorbent/PDMS-like sampler) deployed *in situ* or near-*in situ* to avoid systematically missing neutral and amphiphilic lipids; time-resolved collections, ranging from minutes to hours, are coupled with explicit stress control—such as root integrity or physiological markers and matched “sham” controls, to minimize sampling-induced artifacts; resulting outputs enable quantification of lipid classes (MG/DG/TG, free fatty acids/oxylipins, sterol/sphingolipid-linked features) alongside canonical QS ligands [AHL/diffusible signal factor (DSF)] and oxidation/degradation products; (b) microcosm assays that assign mechanism using reporters and mutants; structured microcosms—comprising surface-attached biofilms and well-mixed planktonic controls—are inoculated with defined strains or synthetic communities carrying QS reporters, along with paired receptor and signal mutants; mechanism-specific perturbations are used to distinguish the four control nodes: receptor mutants and competitive ligand panels probe mimicry/antagonism; lactonase or acylase treatments and ligand half-life assays assess quorum quenching; comparison between biofilm and planktonic growth reveals membrane or microenvironment-mediated effects; and pulses of neutral lipid (MG/DG/TG) together with lipid-access blockade, test the lipid gate; readouts include ligand pools, reporter outputs, and QS regulon expression; (c) compartmented root-hypha systems with spatial lipid mapping and QS readouts; split-compartment designs physically separate roots from AM fungal extraradical hyphae, creating hyphal-only compartments to test whether lipid transfer and hyphal microhabitats modulate QS landscapes; spatially resolved sampling along rhizoplane and hyphoplane gradients is paired with interface-resolved lipid mapping and QS-sensitive readouts such as reporters or metatranscriptomic signatures, thereby enabling causal links between lipid gradients, QS state, and microbial social phenotypes.

The four-node framework also clarifies how each proposed mechanism can be tested experimentally. Node A requires experiments that distinguish altered signal interpretation from unchanged ligand pools, e.g. by combining QS reporters with receptor mutants and competitive ligand assays. Node B requires direct measurements of ligand abundance, ligand half-life, and degradation products in order to separate true quorum quenching or signal protection from secondary effects of growth inhibition. Node C requires spatially resolved measurements of ligand partitioning and interface-associated lipid composition to determine how membranes, vesicles, and biofilm-associated phases reshape signal retention and transport. Node D requires ecological tests in which lipid-rich microsites, local biomass, and QS activation are tracked together in structured populations, allowing resource-enabled threshold crossing to be separated from receptor-level or ligand-level effects (Fig. 2 and Tables 2 and 3).

Several broader priorities follow from this framework. First, the lipid fraction of rhizodeposition should be quantified more systematically across plant functional types, developmental stages, and soil environments using standardized approaches that recover hydrophobic as well as aqueous exudate fractions [27, 28]. Second, the mechanisms governing lipid release need to be resolved more clearly, including the balance between active export and passive release, and the extent to which host-derived lipids are redistributed or transformed across rhizoplane and hyphosphere interfaces. Third, the reciprocal dimension of the framework remains largely unresolved: microbial signals and microbial community composition can remodel host exudation chemistry, but whether the hydrophobic component of these changes feeds back on QS through the four nodes described here remains an open question.

At the community level, the most informative next step will be to test the framework in compartmented systems that allow direct comparison of root- and hypha-associated surface microhabitats [27, 28]. Such designs should help determine whether AM fungal lipid transfer generates reproducible communication hotspots along extra-radical hyphae, whether lipid-enriched microsites promote QS threshold crossing in surface-associated populations, and whether node-specific responses differ systematically between root- and hypha-associated interfaces. More broadly, the minimal causal models summarized in Fig. 2 provide a practical basis for moving from descriptive associations toward mechanistic discrimination across the four nodes.

If these four node-specific mechanisms prove reproducible, they may offer entry points for translational work on lipid-based anti-virulence strategies, host breeding or engineering approaches that reshape exudate composition, and synthetic microbial communities designed to respond predictably to lipid-defined microhabitats. The key challenge will be to distinguish beneficial modulation from “QS hijacking,” in which the same lipid environments instead enhance pathogen persistence, biofilm formation, or virulence. For that reason, the most useful outcome of this framework may be not a single unifying model but a node-based way of asking when, where, and how lipid-rich interfaces make microbial communication more likely, more stable, or more ecologically consequential.

## Data Availability

Data sharing is not applicable to this article as no datasets were generated or analyzed in this review manuscript.
